# Building trust through quality of service mediated with satisfaction to acceptors of family planning in the province of South Kalimantan

**DOI:** 10.1016/j.heliyon.2023.e13142

**Published:** 2023-01-21

**Authors:** Misransyah, Semuel Risal, Mukhlan Khariry, Cici Asmawatiy, Riki Welly Saputra, Sri Sulandjari, Roosganda Elizabeth

**Affiliations:** a STIA Bina Banua Banjarmasin, South Kalimantan, Indonesia; b Universitas Kristen Satya Wacana, Salatiga, Indonesia; c Badan Riset dan Inovasi Nasional (BRIN), Indonesia

**Keywords:** Family planning acceptor, Family planning officer, Satisfaction, Trust, Servqual

## Abstract

This study aims to analyze the effect of service quality on the satisfaction of Long-Term Contraception Method family planning acceptors, analyze the effect of family planning service quality on the trust of Long-Term Contraception Method family planning acceptors, analyzing the effect of acceptor satisfaction on trust, and analyzing the effect of service quality on trust which is mediated by the satisfaction of couples of Long-Term Contraception Method acceptors. So that it can be a reference in making future family planning programs so that it is right on target and can control the rate of population growth. This study uses a quantitative approach, namely research that emphasizes analysis of numerical data obtained by statistical methods to test hypotheses so that the significance of the relationship between the variables studied is obtained. The number of samples in this study was 120 respondents in data collection using multistage sampling and simple random sampling techniques, data analysis techniques using path analysis using the SEM-PLS program. The results showed that the variable quality of family planning officers had a positive and significant effect on the satisfaction of family planning acceptors, the variable of service quality of family planning officers had a positive and significant effect on the trust of family planning acceptors, the variable of satisfaction with family planning acceptors had a positive and significant effect on the trust of family planning acceptors, and the variable of service quality of family planning officers. positive and significant effect on the trust of family planning acceptors mediated by the satisfaction of family planning acceptors using Long-Term Contraception Method.

## 1 Introduction

The population problem facing the world today is the large amount of growth and uneven distribution, as well as the country of Indonesia which has high growth with the fourth largest population in the world with a population growth rate of 1,17% per year. Total fertility rate Indonesia is 2.1 in 2022 this condition makes the target of reducing the population growth rate unattainable. If population growth is not matched by an increase in the quality of the population, then this can lead to a low Human Development Index (HDI) [1].

In such conditions the Family Development, Population, and Family Planning program or known as the “*Bangga Kencana*” which was echoed by the National Population and Family Planning Agency in 2020 which is closer to the millennial generation needs to be given more attention. “Planning is Cool” replaces the old slogan, “Two Children are Enough”, to strengthen Indonesian families and empower women's roles in building Indonesian families. The National Population and Family Planning Agency makes and implements various strategies and policies that accommodate the “Nawa Cita” development agenda and mental revolution starting from the family strategy, together with all sectors supporting the implementation of the family planning program for Couples of Childbearing Ages, which can be accessed by following the national health insurance. In addition, the government's commitment is also realized by allocating special funds in the form of operational facilities and infrastructure for family planning programs starting from 2008 which is now expected to help solve the problem of decentralization, especially with limited resources for the implementation of “*Bangga Kencana”* at the regional level and field lines [2].

The total population in South Kalimantan early in 2018 was 4,055,479 people, based on data from the Banjarmasin City, Central Bureau of Statistics about data on Couples of Childbearing Ages in South Kalimantan Province from 2016 to 2019 there was an increase from year to year. In 2017 the number of Couples of Childbearing Ages in Kalimantan Province South was as many as 688,203, then in 2018 there was an increase of 742,883 people and in 2019 there were 773,489 people in the number of Couples of Childbearing Ages [3]. Based on the explanation and available data that South Kalimantan Province According to the head of the South Kalimantan the National Population and Family Planning Agency, the number of Long-Term Contraceptive Method users in South Kalimantan is still very low compared to other provinces, this is the cause of the population explosion in South Kalimantan. In line with what was stated by the Director of Family Planning Participation in the Private Line of the National Population and Family Planning Agency, he said that until 2021 the use of long-term contraceptives in Indonesia is still a concern [4]. On the other hand, the number of health workers is lacking, especially in hard-to-reach areas so in those areas family planning services are not sufficient. But even in big cities, there are still many family planning failures, to be able to provide a solution so that failure can be minimized to increase and maintain the number of family planning participants, especially for Couples of Childbearing Ages, it is directed to use the Long-Term Contraceptive Method [5]. In this case, what is needed is to build the trust of couples of childbearing ages that by using family planning, they will be able to create a happy and prosperous family [6].

To build trust in family planning participants, Couples of Childbearing Ages, first make sure about how to make them feel satisfied as family planning participants because the satisfaction of family planning acceptors for Couples of Childbearing Ages will have a beneficial impact on parties who are competent with family planning issues. The fulfillment of consumer needs and desires will make the consumer satisfied, this satisfaction will affect consumer confidence in both the product and the organization. The more satisfied the consumer, the more trust the consumer will be, and vice versa, the more dissatisfied the consumer, the less trust will be [7]. This will determine the survival of the organization. In simple terms, satisfaction can be interpreted as an effort to fulfill something or make something adequate [8]. According to Kotler and Keller, Consumer satisfaction is a person's feelings of pleasure or disappointment arising from comparing the perceived performance of the product (or result) against their expectations [9].

Yusrini Meidita from the results of their research show that Satisfaction has a positive effect on Trust, this states that the higher the level of customer satisfaction, the more customer trust in Shopee products [10]. Similarly, what was done by Woro Utari and Hidayat the results of their research showed that patient satisfaction had a significant effect on patient confidence in PHC Surabaya Hospital [11].

Both trust and satisfaction can be built by providing excellent service, Goetsch and Davis define service quality as a dynamic condition associated with goods, services, people, products, and the environment that meet or exceed expectations [12]. Service Quality is a measure of how well the level of service provided can meet customer expectations or expectations. Its production may or may not be linked to a single physical product.

This study aims to analyze the quality of service will affect trust in long-term contraception. Analyze the effect of service quality trust and satisfaction on acceptor loyalty by taking samples of family planning product users. To achieve the research objectives, the authors analyze using the Structural Equation Modeling method because SEM is able to explain the complex relationship between variables and the direct or indirect effect of one or several variables on other variables.

## 2 Literature review

### 2.1 Concept of quality service

**Fig. 1:**
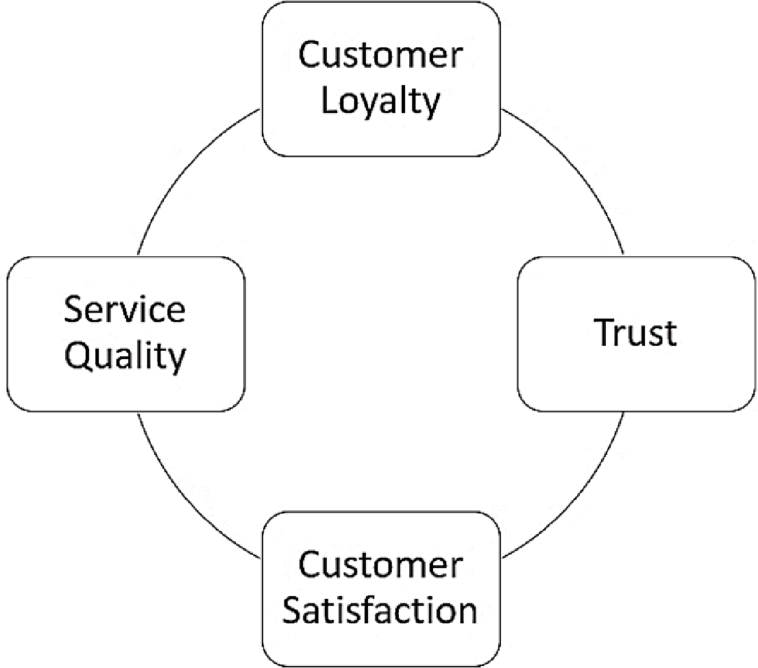
Customer and service relationship.

Trust is a person's willingness to rely on others with whom we have faith. Trust is a mental condition based on a person's situation and social context. When a person makes a decision, he will prefer decisions based on choices from people he can trust more than those who are less trusted [13]. According to Rousseau, trust is a psychological area that is concerned with accepting what is based on expectations of good behavior from others. Consumer trust is defined as the willingness of one party to accept the risk of another party's actions based on the expectation that the other party will act important to the trusting party, regardless of the ability to monitor and control the actions of the trusted party [14]. Trust occurs when a person believes in the reliability and integrity of the person he trusts [15]. This allows the modeler to explicitly obtain measurements in unreliable models, which in theory allows for structural relationships between latent variables to be accurately estimated as shown in Fig. 1.

Research entitled Factors that Determine Quality of Care in Family Planning Services in Africa: A Mixed Evidence Systematic Review There is limited evidence on the factors that determine the quality of care in family planning services in Africa indicating that the quality of care is influenced by many factors. Evidence shows that reducing access barriers and avoiding unnecessary prerequisites for using contraceptive methods are important for improving the quality of services in family planning services. Strategies to improve provider behavior and competence are important. What's more, the strategy minimizes client waiting time and ensures client confidentiality must be applied to ensure the quality of services in family planning services. The weakness of this study is that it is necessary to identify the most important factors associated with quality of care in family planning services in the wider African countries [16].

Research with the title Factors Affecting the Use of Family Planning (KB) in District of Padang Hulu Tebing Tinggi 2018, stated that one of Indonesia's main problems is population growth. This study analyzes the factors that influence the use of family planning in men in the District of Padang Hulu Tebing Tinggi 2018. Based on multivariate results, age and wife support are predictors of male family planning. It is recommended for health workers to increase the promotion of vasectomy services, through the provision of education, knowledge and utilization of family planning vasectomy in the form of counseling and family approaches about the importance of family planning vasectomy and for male family acceptors are expected to be role models for their peers so that others are male. will participate in family planning, especially vasectomy [17].

Study entitled Uptake and impact of in-facility HIV self-testing on PrEP delivery: a pilot study among young women in Kisumu, Kenya. stated that as the scale of pre-exposure prophylaxis (PrEP) increases globally, barriers to the implementation of PrEP remain. At the health care system level, a significant barrier is the complexity of administering PrEP due to the need for regular HIV testing and monitoring of PrEP users. In this pilot evaluation in Kenya, about a third of women taking PrEP chose HIVST over provider-initiated testing, and those who chose HIVST spent less time in the clinic and were generally satisfied with their experience. HIVST in PrEP delivery is feasible and has the potential to simplify PrEP delivery and provide client testing autonomy. The weakness of this study is that it is necessary to explore optimal HIV retesting strategies for PrEP administration, including the use of HIVST in PrEP on a larger scale and in different settings [18].

Study entitled Measuring quality of care at the community level using the contraceptive method plus information index and client-reported experience metrics in Bangladesh. stated that the low rate of continuation of contraception in Bangladesh is a symptom of the poor quality of family planning counseling. Increasing family planning counseling by state public health workers (CHWs) could improve contraceptive continuity. Quality community-based family planning counseling can help address the rising rate of contraceptive discontinuation in Bangladesh. Whereas the MII and MII + scores in this study were low, and FWA showed many misunderstandings, FWA demonstrated strong communication skills that facilitate relationships and trust with clients and their communities [19].

Research entitled Perception of Contraception and Switching Patterns of Contraceptive Methods in Family Planning Acceptor in West Nusa Tenggara, Indonesia. That the perception of family planning acceptors about contraception affects their reasons for choosing to change their contraceptive method. Perceptions among women receiving family planning were found to play an important role in their pattern of switching contraceptive methods. Therefore, fostering a better understanding of contraception through high-quality counseling is needed to increase perceptions and thereby encourage rational, effective and efficient use of contraception [20].

Research entitled Evaluation of intervention processes to improve access to injectable contraceptive services through patent drug vendors in Nigeria: a mixed methods study. stated that the low utilization of modern contraception in many low- and middle-income countries remains a challenge. Patent drug vendors (PMV) operating in the informal health sector have the potential to address this challenge. Outcome evaluation shows increased use of injectable contraceptive services by clients. There is limited information on how and why interventions affect outcomes. PMV continues to play an important role in the provision of family planning services, the need to formalize and scale up these interventions to assist their integral role coupled with multi-faceted initiatives to improve the quality of their services [21].

Research entitled Renewing focus on the quality of family planning services globally. states that every year, as many as 220 million women worldwide have unmet contraceptive needs. As a result, 40% of the 210 million annual pregnancies are unwanted. This study proposes a new approach to improve the standardization and accessibility of family planning service quality assessment tools to simplify the evaluation of family planning service quality. With an easier program evaluation approach, quality improvements can be made more quickly to help increase the use and continuation of contraception to improve the health of women and their families [22].

Research conducted by Yusrini, where the results show that of the five dimensions of Service Quality, there are two dimensions, namely Ease of Use and Layout which have no significant effect on Satisfaction. Woro Utari and Hidayat Hidayat, the results of the study also show that the service quality of the PHC Surabaya hospital is in a good category, but still needs to be improved. Based on the results of data analysis, it is known that service quality has a significant effect on patient trust [11]. Service quality has a significant effect on trust. Research conducted by Setiawan and Ukudi also found that service quality has a positive effect on consumer trust [23].

### 2.2 Approximation of structural method using partial least square (PLS)

SEM processing using the PLS method consists of two general models which are the measuring model (outside model) and the structural model (inside model). The Outer Model shows how the observed variables represent latent variables, where these variables cannot be measured directly so other variables are needed to express them. Meanwhile, the inner model shows the estimated power between these latent variables.

So, it can be said that the model explains the relationship between latent variables. So the model formula can be shown as follows.

1 $$\eta =\beta_{0}+\beta \eta +Τ\xi +\zeta$$

By nature, the PLS method is made for a recursive computation model so that in the relationship between latent variables, each dependent variable η is called a causal chain system. In other words, the set of function equations η will form a latent variable. The equation can be explained as follows:

2 $$\eta_{j}=\sum_{i}\beta_{ji}\eta_{i}+\sum_{b}\gamma_{jb}\xi_{b}+\zeta_{j}$$

Where $\beta_{ji}$ and $\gamma_{jb}$ are trace coefficients that relate the endogenous latent variable (which is deep) η with other endogenous latent variables. The coefficient also has a function to relate the endogenous latent variable and the exogenous latent variable ζ at the distance between i and b, and with $\zeta_{b}$ is the residual form of the inner model at the value $E\left(\zeta_{j}\right)=E\left(\xi_{i}\zeta_{j}\right)=0$.

While the outer model is defined as how each part of the indicator relates to the latent variable. In this study, we used the approach that the external equation model for the indicator section is a simple linear regression equation of the form:

3 $$x_{jk}=\lambda_{jk}\xi_{j}+\varepsilon_{jk}$$

Where $\lambda_{jk}$ is the coefficient of the weighting factor of the relationship between the j th latent variable ($\xi_{j}$) and the kth indicator ($x_{jk}$), and the error of each measuring variable $\varepsilon_{jk}$.

In carrying out the estimation process, the PLS method has three main categories, namely weight estimates, trace estimates, and the average for each location where these parameters are located. We will use this burden to make the value of the latent variable above. The three estimates can be explained as follows.

1 Estimation of weighted value

In the formation of values there are three iterative processes of the linear regression equation from the process of estimating the weight, distance and average of each variable location. The iteration process will stop until we get a stable weight value to calculate the latent variable score which is a linear combination of the indicators.

• *Outer model estimation*

The procedure for calculating the estimation of the outer procedure is, starting with the initial initialization of each latent variable as a linear combination of the indicators shown in the equation: $I_{j}=\sum_{k}\bar{w_{jk}}x_{jk}$.

Where $\bar{w_{jk}}$ is the weight value from the outer model. In this study, we take the definition that the initial weight value is determined based on the value that appears in the first estimate of the latent variable as the aggregate of the indicator values.

• *Inner model estimation*

In estimating the inner model, we also carry out an iterative process on the edge values (connections) between the latent variables in the inner model to obtain the initial value of each latent variable. Where the value of the latent variable is calculated as the sum of the burdens of the paired latent variables. So, the inner estimate is $Z_{j}=\sum_{i:\beta_{ij\neq 0}\;\beta_{ji\neq 0}}e_{ij}l_{i}$.

Where the value of $e_{ij}$ is the internal weight which can be determined from three conditions which are obtained from the centroid value, the factoring or tracking process.

In this study we explain the calculations for each scheme as follows:

4 $$e_{ij}=\left\{ \begin{array}{c} sign\left\{cor\left(I_{i},I_{j}\right)\right\},\xi_{j}\xi_{i}\;adjacent\\ 0,else \end{array}\right.$$

5 $$e_{ij}=\left\{ \begin{array}{c} cor\left(I_{i},I_{j}\right),\xi_{j}\xi_{i}\;adjacent\\ 0,else \end{array}\right.$$

6 $$e_{ij}=cor\left(I_{i},I_{j}\right),if\;\xi_{j}\;is\;denoted\;by\;\xi_{i}\;I_{j}=\sum_{i}e_{ji}l_{i}e_{ij}\;is\;regression\;coefficient\;on\;I_{i}\;and\;I_{j}$$

• Updating outer weight proportionally

o The internal weight $\left(e_{ij}\right)$ in the centroid is the same as the sign of the correlation result between $I_{i}$ and $I_{j}$, where the process centroid is defined by the equation:
o The internal weight $\left(e_{ij}\right)$ in the process factor is the correlation between $I_{i}$ and $I_{j}$ which is taken into account for selecting not only the direction of the sign but also the strength of the path in the inner model.
o Whereas the internal weight in the routing is defined as the weighting of each adjacent latent variable with a reference to whether there are other latent variables that form the antecedent or consequent.

Perform updates on external weights.

Updates on external weights have been completed, the next process is carried out by taking estimates in $Z_{j}$ by considering the observation indicators. This process is processed by considering the $w_{jk}$ variable on $w_{jk}=\left(Z_{j}^{\prime }Z_{j}\right)Z_{j}^{\prime }x_{jk}$.

• Convergence testing method

This process is carried out by iterating n times until the convergent conditions are met. Where the convergent test is carried out by checking the external weight in the k-th step against the weight in the previous step.

2. Estimation of path value

The next process is to perform the calculation process from the path values and weighting $\bar{\beta_{ji}}$ and $\bar{\lambda_{k}}$ in the inner and outer models. In the inner model, the path coefficient is estimated using Ordinary Least Square (OLS) as a multiple linear regression construct on the coefficient between $I_{j}$ and $I_{i}$. With $I_{j}=\sum_{i}\bar{\beta_{ji}}I_{i}$ and $\bar{\beta_{ji}}={\left(I_{i}^{\prime }I_{i}\right)}^{-1}l_{i}^{\prime }I_{j}$. Whereas in the external model, the weighting coefficient is estimated by a process of linear regression by connecting $x_{jk}$ and $I_{j}$. With $x_{jk}=\bar{\lambda_{jk}}I_{j}$ and $\bar{\lambda_{jk}}={\left(I_{j}I_{j}^{\prime }\right)}^{-1}l_{j}^{\prime }\;x_{jk}$.

3. Approximation of variable locations mean

Comparison between the parameters calculated on the properties of the inner model and the outer model (reflective model) will be used to see the two-way (outside-in) position of a latent variable characteristic with its predictors. In other words, we get two parameter locations to be estimated, namely $\beta_{0j}$ as a constant in the inner model and $\lambda_{0jk}$ as a constant in the reflective outer model. So, the new equation can be written as follows:

7 $$E\left(\xi_{j}|\xi_{i}\right)=\beta_{0j}+\sum_{i}\beta_{ji}\xi_{i}\;for\;inner\;model\;and\;E\left(x_{jk}|\xi_{j}\right)=\lambda_{0jk}+\lambda_{jk}\xi_{j}\;for\;outer\;reflective\;model$$

To determine the coefficients $\beta_{0j}$ and $\lambda_{0jk}it\;is\;necessary\;to\;calculate\;the\;mean\;value\;m$ as follows $\bar{\beta_{oj}}=b_{0j}=\bar{m_{j}}-\sum_{i}b_{ji}\bar{m_{i}}$ while parameter $\bar{\lambda_{0jk}}=\bar{x_{jk}}-\bar{\lambda_{jk}}m_{j}$ dan $\bar{\pi_{0j}}=\bar{m_{j}}-\sum_{k}\bar{\pi_{jk}}\bar{x_{k}}$. With mean ${\bar{m}}_{j}=\sum_{k}\bar{w_{jk}}\bar{x_{jk}}$ dan $\bar{\xi_{j}}=I_{j}+\bar{m_{j}}$.

## 3 Research method

The research method used in this research is the quantitative analysis method, namely research that emphasizes analysis of numerical data obtained by statistical methods and is carried out in inferential research or to test hypotheses so that the significance of the relationship between the variables studied is obtained. This study uses Structural Equation Modeling (SEM), which is a statistical technique for testing and estimating causal relationships using a combination of statistical data and qualitative causal assumptions. SEM is a method of multivariate statistical analysis. Processing SEM data is different from processing regression data or path analysis. SEM data processing is more complicated, because SEM is built by measurement models and structural models. In SEM there are 3 activities simultaneously, namely checking the validity and reliability of the instrument (confirmatory factor analysis), testing the relationship model between variables (path analysis), and obtaining a suitable model for prediction (structural model analysis and regression analysis). A complete model basically consists of a measurement model and a structural model or causal model. The measurement model is carried out to produce an assessment of the validity and discriminant validity, while the structural model is a model that describes the hypothesized relationships [24]. The advantage of using SEM is the ability to construct latent variables, namely variables that are not measured directly, in a model of several measured variables, each of which is estimated according to the latent variable.

The approved ethics committee was Dr. Irawanto. Closed questionnaire was used in this study, with answer choices provided. The questions in the questionnaire are:

1. Instructions for filling out the questionnaire
2. Respondent identity: consisting of name, gender, age, education, occupation, contraceptive used, since when the contraceptive was used, number of children
3. Trust of the service quality consist of very dissatisfied, dissatisfied, unsatisfied, satisfied, very satisfied
4. Service quality variables long term contraception method consists of reliability, responsiveness, assurance
5. Family planning acceptor satisfaction variables: fitness, convenience, availability
6. Acceptor's trust variables family planning consisting of: ability, integrity, goodness

The population is a generalization area consisting of objects/subjects that have certain quantities and characteristics determined by the research to be studied and then conclusions are drawn [25]. The population in this study were family planning acceptors for Couples of Childbearing Ages who used the Long-Term Contraception Method in 4 (four districts and cities in South Kalimantan Province). The object of this research was taken based on the representation of urban areas and rural areas, and based on the representation of the highest and lowest acceptors in the use of Long-Term Contraception Method. Based on data obtained from the Feedback Report in December 2019, the Long-Term Contraception Method Acceptors are as follows Table 1:

**Table 1:** Population.

TYPE OF LONG-TERM CONTRACEPTION METHOD	BANJAR BARU	KOTA BARU	TAPIN	HULU SUNGAI TENGAH (HST)
MOW	96	49	17	42
MOP	7	0	5	6
IUD	331	42	21	231
IMPLANT	449	282	204	646
**TOTAL**	**883**	**373**	**247**	**925**

The sample is part or representative of the population under study. It is called a sample if we intend to generalize the results of a sample study. The sample in this study were couples of childbearing age family planning acceptors that use Long-Term Contraception Method. Thus, the size of the sample used is 96 respondents, but to keep the questionnaire accountable, the researchers set the respondent to be 120 respondents. The sampling technique used is random sampling using stratified sampling.

To determine the effect between variables, the analytical method used is the path analysis method to analyze the pattern of relationships between variables. This model is used to determine the direct or indirect effect of a set of independent variables (exogenous) on the dependent variable (endogenous). Data analysis using SEM-PLS software, as the variables in Table 2, Table 3, and Table 4.

**Table 2:** Family service quality variables planning long-term contraceptive methods.

Variables	Indicators
Reliability $X_{1}:\left\{x_{1.1},x_{1.2},x_{1.3}\right\}$	• Counseling services provided by family planning officers are fast and correct $\left(Symbol:x_{1.1}\right)$ • Medical personnel are able to provide reliable and appropriate services $\left(Symbol:x_{1.2}\right)$ • Conditions and acceptor data are carefully checked and recorded $\left(Symbol:x_{1.3}\right)$
Responsiveness $X_{2}:\left\{x_{2.1},x_{2.2},x_{2.3}\right\}$	• The ability of family planning extension officers to respond quickly in dealing with complaints from acceptors before and after service $\left(Symbol:x_{2.1}\right)$ • Family planning instructors provide information in a clear and easy to understand manner $\left(Symbol\;x_{2.2}\right)$ • Family planning service officers are not in a hurry to install contraceptives $\left(Symbol:x_{2.3}\right)$
Assurance $X_{3}:\left\{x_{3.1},x_{3.2}\right\}$	• Guaranteed reliable long term contraceptive method family planning services $\left(Symbol:x_{3.1}\right)$ • The accuracy of the family planning service officer is as desired by the family planning acceptor $\left(Symbol:x_{3.2}\right)$

**Table 3:** Receiver satisfaction variable.

Variable	Explanation of Variables	Indicators
Satisfaction Variable from fitness, easy to access, and wiliness to be acceptor $Y_{1}:\{Y_{1.1},Y_{1.2},Y_{1.3}\text{,}$ $Y_{1.4},Y_{1.5},Y_{1.6},Y_{1.7}\}$	Fitness with customer need	• Family planning services meet my expectations $\left(Symbol:Y_{1.1}\right)$ • The results I received were very satisfying $\left(Symbol:Y_{1.2}\right)$ • This type of contraception is very beneficial $\left(Symbol:Y_{1.3}\right)$
	Easy to access	• Family planning services are not complicated. $\left(Symbol:Y_{1.4}\right)$ • Family planning services don't take long $\left(Symbol:Y_{1.5}\right)$
	Willingness for becoming acceptor	• Family planning acceptors can be served at any time as needed $\left(Symbol:Y_{16}\right)$ • Willingness to give extra information and recommendation to others if necessary $\left(Symbol:Y_{1.7}\right)\text{.}$

**Table 4:** Receiver loyalty variable.

Variable	Explanation variable	Indicator
Loyalty variable which derived from capability of doing service, Integrity, and kindness $Z_{1}:\left\{Z_{1.1},Z_{1.2},Z_{1.3}\text{,}\right\}$ $\left\{Z_{1.4},Z_{1.5},Z_{1.6}\right\}$	Capability of doing service	• The capability of service by the servant officer. $\left(Symbol:Z_{1.1}\right)$ • Effectiveness service and assistance by officers. $\left(Symbol:Z_{1.2}\right)$
	Integrity	• Officer is having good reputation of doing service $\left(Symbol:Z_{1.3}\right)$ • The officers are trusted by their customer of doing service as what they belief. $(Symbol:Z_{1.4}$).
	Kindness by hospitality	• Officers shown hospitality to the customer $\left(Symbol:Z_{1.5}\right)$ • Officers shown good intention to help customers $\left(Symbol:Z_{1.6}\right)$.

### 3.1 The algorithm procedure

Based on theoretical foundation using PLS we define procedure of computing the algorithm as follows.

1. We do preliminary research and preparation on factors that influence service quality of BKKBN (Birth countermeasure agency).
2. We create conceptual model for constructing path diagram. Determination of service quality model of long-term contraceptive program. From our concept, we build hypothesis as follows:

H1 Assurance has relationship with loyalty and satisfaction.

H2 Reliability has relationship with loyalty and satisfaction

H3 Responsiveness has relationship with loyalty and satisfaction

H4 Satisfaction has relationship with loyalty.

3. We evaluate each indicator variable by observing convergent validity, discriminant validity and value of composite reliability.
4. Process estimating the score of latent variables.

**Fig. 2:**
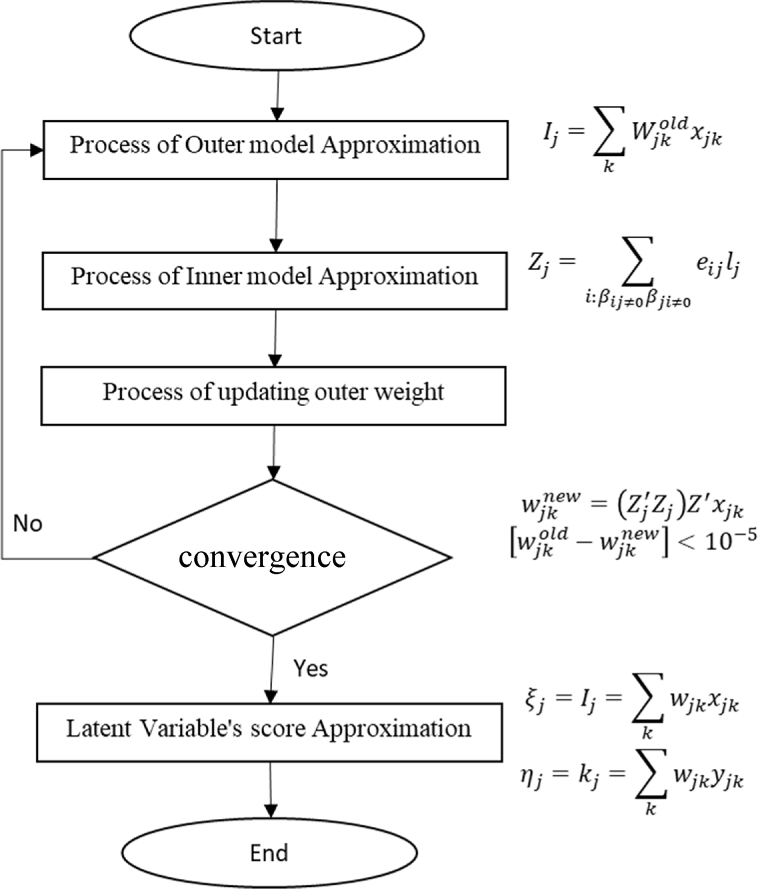
Process iteration of updating latent variable score.

In this research we set the score of exogenous variables $\xi_{j}=I_{j}$ and the endogenous variable is $\eta_{j}=k_{j}$. The process of computation can be seen via process diagram as shown in Fig. 2.

5. Select the best model → do analysis → Result

Request permission is submitted before carrying out a study. The permit application is intended for the Ethics Committee of the College of Administrative Sciences (STIA) Bina Banua Banjarmasin as a place for ethical due diligence. Steps for submission of ethical clearance:

1. Proposing and giving it to the Ethics Committee for the College of Administrative Sciences (STIA) Bina Banua Banjarmasin, as well as giving information and answers to the questions posed to research, does not violate ethical eligibility.
2. An application for an ethical clearance permit is submitted to the Ethics Committee of the College of Administrative Sciences (STIA) Bina Banua Banjarmasin.
3. The research was carried out after receiving a letter of eligibility for ethical clearance issued by the Ethics Committee of the College of Administrative Sciences (STIA) Bina Banua Banjarmasin, signed by the Chairperson of the Ethics Committee, Dr.Irawanto.
4. We also confirm that informed consent was obtained from all participants for experiments.

## 4 Results and discussion

### 4.1 Main result of outer and inner model convergent validity

**Fig. 3:**
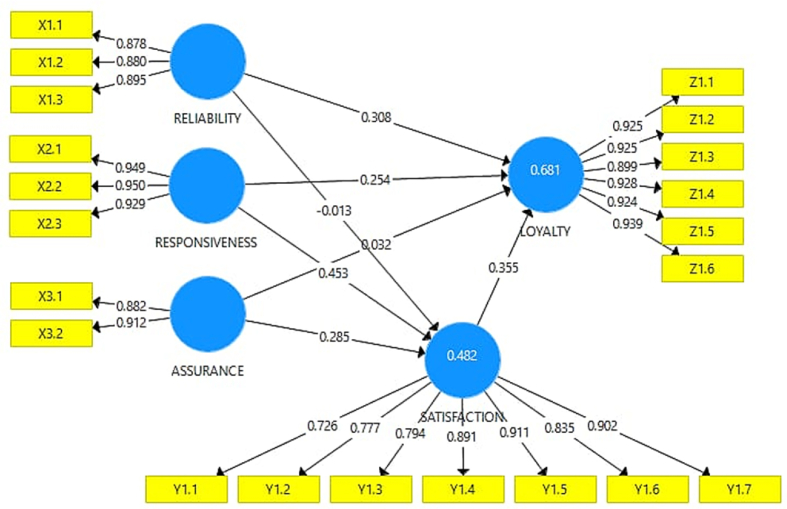
Outer model (cross-weighting).

**Fig. 4:**
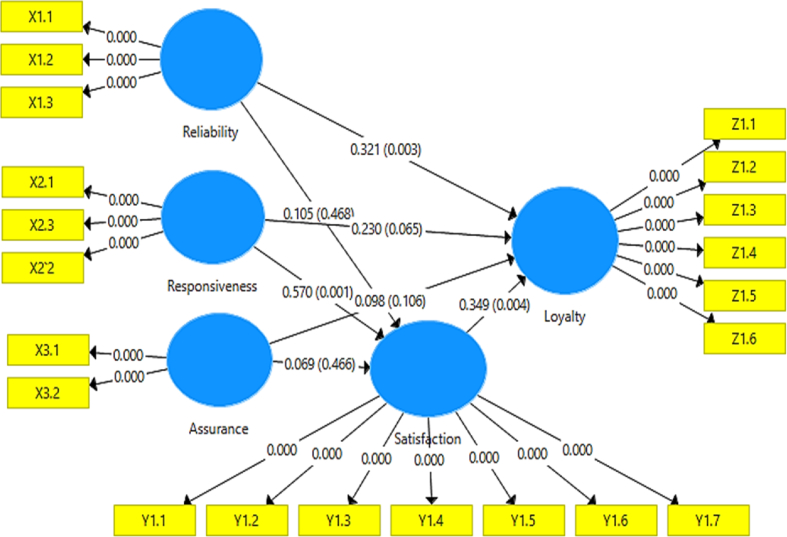
Inner model.

The weight factor results from the computation of SEM algorithm (Fig. 2) is resulted by three indicators of weighted schemes process, which are centroid, factor and path method. They have been constructed by 21 indicators which is spread based on their latent variables. However, in our research we use 5 latent variables, namely reliability, responsiveness, assurance, loyalty and satisfaction. The design of the measurement model (outer model) in PLS is very important because it is related to whether the indicator is reflective or formative. The measurement Outer Model in this study is as shown in Fig. 3. On the other hand, the design of the structural model of the relationship between latent variables in PLS (inner model) is based on the formulation of the problem or research hypothesis. Based on the result of inner model in Fig. 4.

Judging from the result above, the measurement of the accuracy of the instrument used in the study, to find out whether the instrument used is valid or not, can be seen on the line connecting the variables. latent with the observed variable, if the relationship number shows above 0.50 then the relationship can be said to be valid so that the questionnaire can be used in collecting data in the field, and if on the contrary, the number is below 0.50 it can be said that the indicator is invalid.

Taking into account the results of these calculations from 119 returned questionnaires, it can be concluded that the three variables, namely the Service Quality Variables for family planning personnel/officers such as reliability, assurance and responsiveness, Acceptor Satisfaction Variables, and Family Planning Acceptor Trust variables, it can be said that all statement items are valid, and for the measurement of construct reliability the results areas in the following Table 5:

**Table 5:** Construction reliability.

	Cronbach's Alpha	rho_A	Composite Reliability	Average Extracted Variance (AVE)
Assurance	0.761	0.768	0.893	0.807
Loyalty	0.965	0.967	0.972	0.852
Reliability	0.866	0.911	0.915	0.782
Responsiveness	0.937	0.938	0.960	0.888
Satisfaction	0.929	0.949	0.942	0.699

From the table above, of the five variables used in this study, both as measured by Cronbach's Alpha and Composite Reliability, the numbers are above 0.60, so the instrument is reliable, as shown in Table 6.

**Table 6:** Direct relationship (direct effect).

	Original Sample (O)	Sample Average (M)	Standard Deviation (STDEV)	T Statistics (|O/STDEV |)	P Values
Assurance - > Loyalty	0.098	0.095	0.060	1.617	**0.106**
Assurance - > Satisfaction	0.069	0.078	0.094	0.730	**0.466**
Reliability - > Loyalty	0.321	0.320	0.109	2,955	**0.003**
Reliability - > Satisfaction	0.105	0.118	0.144	0.726	**0.468**
Responsiveness - > Loyalty	0.230	0.217	0.124	1,852	**0.065**
Responsiveness - > Satisfaction	0.570	0.558	0.166	3.427	**0.001**
Satisfaction - > Loyalty	0.349	0.370	0.119	2,925	**0.004**

### 4.2 Hypothesis testing

We stated to find out whether or not the relationship between variables has an effect, see the following table the results of calculations using SEM PLS software as shown in Table 6. Based on the table above, the calculation results for the first hypothesis which reads that the quality of family planning services such as reliability, responsiveness, and assurance is only the responsiveness variable that has a positive and significant influence on the satisfaction of family planning acceptors. where the count is 3.427, with a significance level of 0.001, and this result is compared with the standard used, namely the T value > 1.96 and the P-value <0.05, while the reliability and assurance variables have an effect but are not significant for the hypothesis.

The second statement states that the quality of family planning services such as reliability, responsiveness, and assurance is only the reliability variable that has a positive and significant impact on the trustworthiness of family planning acceptors using Long-Term Contraception Method. where the count is 2,955, with a significance level of 0.003, and this result is compared with the standard used, namely the T value > 1.96 and the P-value <0.05, while the variable responsiveness and assurance have an effect but not significant. The third hypothesis which states that the satisfaction of Long-Term Contraception Method family planning acceptors has a positive and significant effect on the trust of Long-Term Contraception Method family planning acceptors is proven to be true. where the count is 2,925, with a significance level of 0.004, and this result is compared with the standard used, namely the T value > 1.96 and the P-value <0.05, as in Table 7.

**Table 7:** Indirect effects.

	Original Sample (O)	Sample Average (M)	Standard Deviation (STDEV)	T Statistics (|O/STDEV |)	P Values
Assurance - > Loyalty	0.024	0.032	0.040	0.596	**0.551**
Assurance - > Satisfaction					
Reliability - > Loyalty	0.037	0.046	0.060	0.614	**0.540**
Reliability - > Satisfaction					
Responsiveness - > Loyalty	0.199	0.203	0.087	2.279	**0.023**
Responsiveness - > Satisfaction					
Satisfaction - > Loyalty					

Meanwhile and for the fourth hypothesis which states that the quality of family planning services such as reliability, responsiveness, and assurance, it turns out that only the responsiveness variable has a positive and significant influence on the trust of family planning acceptors for Long-Term Contraception Method users mediated by acceptor satisfaction. where the count is 2.279, with a significance level of 0.023, and this result is compared with the standard used, namely T value > 1.96 and P-value <0.05.

For the indirect effect, it turns out that from the 3 independent variables used only one shows a significant effect, namely responsiveness has a positive and significant effect on loyalty mediated by family planning acceptor satisfaction, while the two variables, namely reliability, and assurance, have a positive but not significant effect.

Evaluation of the structural model (inner model).

In Table 8, it appears that the coefficient of determination using the value of R^2^ shows that the effect of endogenous construct variations can be explained by the variables that influence it on the hypothesis. The value of R^2^ for the Satisfaction variable is 0.466 or shows that the acceptor satisfaction variable can be explained by 46.60% by the service quality variable. This means showing the relationship between service quality such as reliability, assurance, and responsiveness in explaining acceptor satisfaction. In addition, the R^2^ value for the trust variable is 0.688, meaning that the contribution given ^by^ the service quality variable and acceptor satisfaction to trust is 68.80%.

**Table 8:** Values of R2 and R2 Adjusted.

	R Square	Adjusted R Square
Loyalty	0.688	0.677
Satisfaction	0.466	0.452

The following for testing the inner model can be done by looking at the value of Q^2^ (predictive relevance) The value of Q^2^ ^can^ be used with the formulation:

$$Q^{2}=1\;–\;\left(1-R^{2}1\right)\;\left(1-R^{2}2\right)$$

$$Q^{2}=1\;–\;\left(1\;–\;0.466\right)\;\left(1\;–\;0.688\right)$$

$$Q^{2}=1\;–\;\left(0.534\right)\;\left(0.312\right)$$

Q2=0.8333

That is, the exogenous latent variable is simultaneously able to explain the variability of the endogenous latent variable of 0.833 or 83.30%. Meanwhile, the remaining 16.70% is explained by other variables not included in the model. Thus, the three independent variables can make a big contribution. So, the model is feasible to use.

Based on the results of testing the hypothesis above, it shows that service quality has a significant influence in shaping customer satisfaction. While customer satisfaction is one of the variables that build customer loyalty in addition to the variables of trust and service quality itself. From this statement, it can be stated that to maintain the acceptors of the South Kalimantan Long-Term Contraceptive Method, it can be realized by paying attention to service quality and customer satisfaction while not forgetting the trust variable which also has an effect.

## 5 Discussion

The relationship between the service quality of family planning officers and the satisfaction of family planning acceptors, The effect of service quality on family planning acceptor satisfaction. The results of the study show that the service quality of family planning officers has a positive and significant influence on the satisfaction of Long-Term Contraception Method acceptors in four districts/cities in South Kalimantan Province. This can be interpreted that the better the service quality of family planning officers based on the perception of Long-Term Contraception Method users, the satisfaction of the acceptors formed will also be good or high. Based on this research, the service quality of family planning officers has an important role in the formation of a general perception that will affect the level of satisfaction of family planning acceptors using Long-Term Contraception Method.

The relationship between service quality and satisfaction is widely documented in the literature, especially in marketing, the relationship is both theoretically and empirically positive as has been investigated. When the service provided can meet or exceed the expectations or expectations of customers, the customer is satisfied. Quality of service as a measure of how well the level of service provided is under customer expectations”. Quality of service is needed so that customer satisfaction with good service is met [26].

The results of this study showed that good service quality will result in high customer satisfaction which can increase customer loyalty [27]. Akbar, Arifin and Sunarti, also revealed the same thing, namely service quality from five dimensions: tangibles, reliability, responsiveness, assurance, empathy has a positive impact on customer satisfaction [28], and many research results that have different sample backgrounds have proven that service quality has a positive influence on customer satisfaction [29,30].

### 5.1 The influence of service quality on trust

The results showed that the service quality of family planning officers had a positive and significant influence on the trust of family planning acceptors. This can be interpreted that the better the quality of service from family planning officers, especially services from family planning officers, the trust of family planning acceptors will also be good or high. Based on this research, the service quality of family planning officers has an important role in the formation of a general perception that will affect the level of trust of Long-Term Contraception Method users. Therefore, it can be concluded that the items on the variable of service quality of officers and acceptor trust can be accepted by respondents, namely users of Long-Term Contraception.

The results of this study support the research of Chan and Chiu which shows that there is a positive relationship between service quality and consumer trust [31].

### 5.2 The effect of family planning acceptor satisfaction on acceptor trust

The results showed that the satisfaction of family planning acceptors had a positive and significant effect on the trust of family planning acceptors. This can be interpreted that the higher the satisfaction of the acceptor in terms of receiving services from family planning officers, the level of trust of the acceptor formed will also be high. Based on this research, the satisfaction of family planning acceptors has an important role in the formation of a sense of security and trust in the services of family planning officers which will affect the level of trust of acceptors towards family planning officers themselves.

The results of this study shows that there is a positive relationship between service quality and consumer trust. Thus, consumers who believe in the company will become regular customers because of the guarantee of good service quality.

### 5.3 The effect of satisfaction on acceptor trust is mediated by acceptor satisfaction

After knowing that there is an effect of acceptor satisfaction on acceptor trust and the influence of family planning officer service quality on acceptor satisfaction and trust, then a mediation analysis is carried out, namely an analysis of the effect of family planning officer service quality on acceptor trust through acceptor satisfaction. The results showed that the service quality of family planning officers had a positive and significant influence on the trust of family planning acceptors mediated by acceptor satisfaction. This can be interpreted that the higher the trust of the acceptor in terms of receiving services from family planning officers mediated by the satisfaction of the acceptor, the higher the level of trust of the acceptor formed loyalty and they ready to influence others to accept becoming acceptor.

The results of this study support the research of Jason M. Carpenter and Ann Fairhurst. From the results of this study, it can be seen that consumer satisfaction can be used as a mediating variable. His research uses utilitarian and hedonic shopping benefits, customer satisfaction, and word of mouth communication variables. From the results of this study, it can be seen that consumer satisfaction can be used as a mediating variable between utilitarian and hedonic shopping benefits, with customer loyalty [32].

But the results of this study are different from the research of Kurniawan, where the results of data analysis show that consumer satisfaction does not mediate the effect of perceived quality on consumer loyalty. This means that the existence of consumer satisfaction does not strengthen the influence of perceived quality on consumer loyalty [33]. This difference is due to the products studied in previous studies are products in the form of goods.

## 6 Conclusion

Based on the analysis of the research results and the discussion in the previous chapter, several conclusions can be drawn to provide answers to the hypotheses put forward. The first finding is we found a positive and significant influence on the service quality of officers with the satisfaction of Long-Term Contraception Method user acceptors. There is a positive and significant effect of acceptor satisfaction with the trust of acceptors of Long-Term Contraception Method users in South Kalimantan Province. This shows that if the quality-of-service increases, the level of satisfaction and trust of family planning acceptors will also increase, as well as if the satisfaction of family planning acceptors increases, the trust of acceptors for family planning will increase.

Moreover, we also found that there is a positive and significant effect of service quality on the trust of Long-Term Contraception Method users through acceptor satisfaction in Prov. South Borneo. This means that the better the quality of services provided by the officers through the satisfaction of family planning acceptors, the higher the trust of family planning acceptors using Long-Term Contraception Method in South Kalimantan Province.

### Ethical statement

Hereby, We as authors (Misransyah, Semuel Risal, Mukhlan Khariry, Cici Asmawatiy, Riki Welly Saputra, Sri Sulandjari, and Roosganda Elizabeth) consciously assure that for the manuscript titled “BUILDING TRUST THROUGH QUALITY OF SERVICE MEDIATED WITH SATISFACTION TO ACCEPTORS OF FAMILY PLANNING IN THE PROVINCE OF SOUTH KALIMANTAN” the following is fulfilled. 1) This material is the authors' own original work, which has not been previously published elsewhere. 2) The paper is not currently being considered for publication elsewhere.

### Author contribution statement

Misransyah: Conceived and designed the experiments; Analyzed and interpreted the data; Contributed reagents, materials, analysis tools or data; Wrote the paper.

Semuel Risal: Conceived and designed the experiments; Analyzed and interpreted the data; Contributed reagents, materials, analysis tools or data; Wrote the paper.

Mukhlan Khariry: Performed the experiments; Wrote the paper.

Cici Asmawatiy: Performed the experiments; Wrote the paper.

Riki Welly Saputra: Conceived and designed the experiments; Analyzed and interpreted the data; Contributed reagents, materials, analysis tools or data.

Sri Sulandjari: Performed the experiments; Analyzed and interpreted the data; Contributed reagents, materials, analysis tools or data; Wrote the paper.

Roosganda Elizabeth: Performed the experiments; Analyzed and interpreted the data; Wrote the paper.

### Funding statement

This research did not receive any specific grant from funding agencies in the public, commercial, or not-for-profit sectors.

### Data availability statement

Data will be made available on request.

### Declaration of interest’s statement

The authors declare no conflict of interest.
